# PreHospital Ambulance Stroke Test - pilot study of a novel stroke test

**DOI:** 10.1186/s13049-017-0377-x

**Published:** 2017-04-11

**Authors:** Gunnar Andsberg, Magnus Esbjörnsson, Arne Olofsson, Arne Lindgren, Bo Norrving, Mia von Euler

**Affiliations:** 1 Department of Rehabilitation Medicine and Neurology, Lund University, Skane University Hospital, Lund, SE-221 85 Sweden; 2 Department of Medicine, Hässleholm, Sweden; 3 Region Skåne Prehospital Unit, Lund, Sweden; 4 Department of Clinical Science and Education, Södersjukhuset, Karolinska Institutet, Karolinska Institutet Stroke Research Network at Södersjukhuset, Stockholm, Sweden

**Keywords:** Cerebrovascular diseases, Strokes, TIA, Treatment, Prehospital, Stroke Scale

## Abstract

**Background:**

There is a need for a prehospital stroke test that in addition to high sensitivity for stroke, also is able to communicate stroke severity similar to the National Institute of Health Stroke Scale (NIHSS).

**Methods:**

The PreHospital Ambulance Stroke Test (PreHAST), an eight item test based on NIHSS, which scores stroke severity from 0–19 points, was designed and adapted for the ambulance services. In the pilot study the ambulance nurses used PreHAST to assess patients with suspected stroke in the prehospital setting. Regardless of the results after PreHAST testing the patients were triaged with a provisional stroke diagnosis. The PreHAST scores were compared with the final diagnosis and the ability to differentiate stroke and transient ischemic attacks (TIA) with ongoing symptoms at evaluation from non-stroke patients was analysed.

**Results:**

69 patients were included in the study, 26 had stroke/TIA and 43 other diagnoses. All stroke/TIA patients were identified by PreHAST (sensitivity 100% (95% CI; 87-100%)). The specificity increased with higher PreHAST scores and the discriminative capacity for PreHAST for different cut off values showed an area under the curve of 0.77 (95%CI; 0.66-0.88) in the receiver operating characteristic (ROC) analysis.

**Discussion:**

PreHAST is designed for high sensitivity, screening for a broad range of stroke symptoms including most key components of NIHSS. The promising sensitivity between 87 and 100% in our study has to be confirmed in a larger study also including multiple centres. Higher PreHAST scores implied more typical patterns of stroke and accordingly the proportion of stroke mimics decrease with higher scores. However, also stroke mimics with epilepsy/seizure and patients with deficit after prior stroke could show higher PreHAST scores. Other prehospital stroke tests that evaluate stroke severity have been designed with the main purpose to screen for large vessel occlusion. The advantage of PreHAST is the dual purpose not only to evaluate stroke severity but also to screen for stroke in general.

**Conclusions:**

PreHAST is a new screening test of stroke adapted for ambulance services that in addition to high sensitivity for stroke, provides a grading system with increasing specificity with higher scores.

## Background

With new reperfusion treatments for stroke, i.e. thrombectomy, fast identification of stroke and stroke severity have become crucial [1]. Prehospital identification and pre notification of stroke minimize time loss to acute stroke treatments [2] instigating a need for a comprehensive test of typical stroke symptoms for ambulance services [3]. The prehospital tests for stroke screening with the highest reported sensitivity are the Face Arm Speech Test (FAST) [4, 5] and the Cincinnati Prehospital Stroke Scale (CPSS) [3, 6]. A concern is that these tests restrict evaluation to unilateral facial palsy, arm paresis and speech disturbances, and may miss patients with other disabling stroke symptoms. Furthermore, a modern prehospital stroke test should, in addition to high sensitivity for stroke, also be able to identify patients with severe stroke symptoms and thus the most likely candidates for thrombectomy [7].

## Methods

### The basis of the PreHAST design

The PreHAST was designed to screen for common stroke symptoms and grade severity, similarly to the NIHSS [8]. Evaluation of neurological signs with high prevalence in stroke as compared to stroke mimics in prehospital screening, i.e. facial palsy, arm paresis, dysphasia/dysarthria, hemianopia and sensory loss of arm and leg [9], were included. Beyond evaluation of common stroke symptoms, items which in multivariate analysis predict main arterial vessel occlusion were also incorporated, viz. the sub item “questions” in level of consciousness (LOC) evaluation, gaze, leg paresis and neglect [10].

To make the test more feasible in prehospital-paramedics setting than NIHSS the following simplifications were made. Simultaneous testing of right and left side for visual field and sensory items should ensure sensitivity also in patients with neglect. By only allowing verbal instructions in the commands item this indirectly also tests for sensory (Wernicke’s) aphasia. In the speech/language item “dysarthria” is not distinguished from “aphasia”. This resulted in an eight item test (Table 1), which scores stroke severity from 0–19 points. Furthermore, PreHAST is intended for use only in conscious patients*,* i.e. alert or aroused by minor stimulation.

**Table 1:** The PreHospital Ambulance Stroke Test

1. Commands Only verbal instruction: Close your eyes! Grip your hand! (non-paretic side)	0 - Both correct	Score	
	2 – One or none correct		
2. Eye position Observe if the patient “gaze” at one side without purpose	0 - Normal	Score	
	2 - The patient”gaze” preferably or only at one side		
3. Visual field Look the patient straight in the eyes and wave on either side simultaneously. Ask the patient to point at the hand or hands waving.	0 - Normal	Score	
	2 – Apprehends only waving on one side		
4. Facial palsy Ask the patient to smile	0 – Normal	Score	
	1 – One corner of the mouth hanging		
5. Arm paresis Laying or sitting position. One arm at a time. Start with the best arm. Lift arm 45° and ask to hold 10 s. Count down verbally. Assist if doesn’t manage to lift herself. If inability to hold is caused by pain score 0.	0 – Holds for 10 s	Right	Left
		Score	Score
	1 – Drifts but does not reach bed in 10 s		
	2 – Drifts and reach bed in 10 s or falls immediately		
6. Leg paresis Laying or sitting position. One leg at a time. Start with the best leg. Lift leg 30° and ask to hold 5 s. Count down verbally. Assist if doesn’t manage to lift herself. If inability to hold is caused by pain score 0.	0 – Holds for 5 s	Right	Left
		Score	Score
	1 – Drifts but does not reach bed in 5 s		
	2 - Drifts and reach bed in 5 s or falls immediately		
7. Sensory (pain) Pinch the bend of the arms and legs, respectively. Pinch simultaneously at left and right side. Ask if she can feel the pinch in the same way on both sides.	0 - Normal	Score	
	1 – Apprehends less or different on one side		
	2 – Apprehends only on one side		
8. Speech and language Note in the course of conversation. If uncertain, ask patient to repeat a simple sentence, such as “the weather is pretty today”. Score 2, if mute.	0 - Normal	Score	
	1 –Slight or moderate dysarthria or aphasia. Communication possible.		
	2 – Severe dysartria or aphasia. Communication not intelligible.		
PreHAST score	Sum up total score (0–19 points)	Total score	

### Use of PreHAST in a prehospital setting

A pilot study of all patients assessed with PreHAST was performed January 9 to May 23, 2014, in the ambulance district, staffed by 43 ambulance nurses for service around the clock for the community hospital of Hässleholm. The hospital catchment area is 2 068 km^2^, population 70 000 and stroke incidence approximately 305/100,000/year.

Before the study period all ambulance nurses received a 4-h education program, covering basic stroke knowledge and assessment and grading of stroke symptoms according to PreHAST. The education program included practical PreHAST training in pairs, where each ambulance nurse performed the PreHAST items under supervision and proper execution. During the study an instruction video for PreHAST was available on YouTube [11].

During the study period neurological assessment with PreHAST was done if stroke was suspected, defined as sudden onset of focal neurological symptoms/signs, in conscious patients above 18 years. Regardless of test result, the ambulance teams transported the patients according to the guidelines for suspected stroke. Informed consent was obtained the following working day. Patients with stupor or coma are in our region transported according to the rapid emergency triage and treatment system (RETTS) [12, 13] as “unstable vital signs” with highest priority for further acute evaluation of cause at the emergency room.

Two stroke physicians (GA, ME), blinded to the PreHAST scores, independently reviewed the medical records of the patients, for current diagnosis. If the reviewers disagreed after evaluation of history, clinical and radiological findings, a third evaluator (AL) adjudicated the final diagnosis. The diagnoses were divided in “stroke/TIA” with ongoing symptoms at ambulance evaluation and “Non-stroke” diagnosis (stroke mimics). A time based definition for TIA was used, thus for temporary focal neurological symptoms or amaurosis fugax not lasting more than 24 h irrespective of stroke treatment, with no apparent cause other than cerebral, retinal or spinal ischemia. The TIA patients included in the study were required to have ongoing symptoms when evaluated by the ambulance staff. Stroke was defined as rapidly developing clinical signs of focal or global disturbance of cerebral function, with symptoms lasting 24 h or longer or leading to death, with no other apparent cause than vascular. Information on treatment with intravenous thrombolysis (IVT) was collected. Patients with deficit in any PreHAST item (score 1–19) were defined as PreHAST positive. After the study was completed, the ambulance staff received a questionnaire about ease/difficulties with and time to perform PreHAST.

### Correlation analysis between PreHAST and NIHSS

A theoretical simulation of correlation between NIHSS and PreHAST was made based on a registry of thrombolysis of 132 patients where complete NIHSS scores were recorded.

### Statistical analyses

The accuracy of PreHAST to identify stroke/TIA with ongoing symptoms was analyzed by calculating sensitivity, specificity and positive and negative predictive values. The discriminative capacity of the full range of the PreHAST scores was analyzed for different cut-off values using the area under the receiver operating characteristic (ROC) curve.

A descriptive analysis of the patients with Non-stroke diagnoses with positive scores after PreHAST evaluation was performed. This group of patients is of special interest because these by the screening instrument falsely identified patients are at risk of time delay to correct treatment for other conditions than stroke.

The NIHSS and the simulated PreHAST scores were plotted and correlation coefficient was calculated using Excel® and the 95% confidence interval was calculated using VasserStats.net.

## Results

In all, 78 patients were assessed with PreHAST. Nine patients were excluded; in five informed consent was not obtained; in two PreHAST was not possible to perform due to agitation and ongoing epileptic seizure, respectively, and two patients had no symptoms at ambulance arrival.

**Fig. 1:**
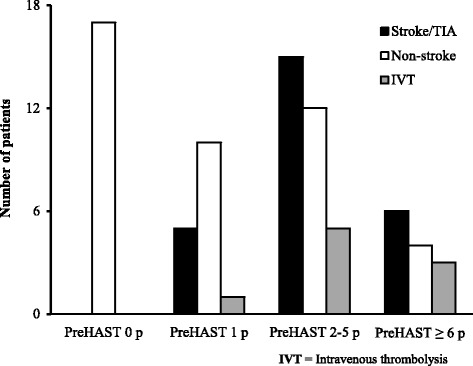
Distribution of the PreHAST scores in the cohort

Of the remaining 69 patients, the final diagnosis was stroke/TIA for 26 and other diagnosis for 43 patients (Non-stroke) (Table 2). All Stroke/TIA were identified by PreHAST, (Table 3). Nine patients received IVT, all of which were identified by PreHAST (Fig. 1).

**Table 2:** Diagnoses for the patients in the Stroke/TIA, and Non-stroke groups tabulated for results after PreHAST

		PreHAST positive	PreHAST negative
Stroke/TIA	Hemorrhagic stroke	1	0
	Ischemic stroke	18	0
	TIA	7	0
Non-stroke	Epilepsy/seizure	7	1
	Late effect after stroke	7	0
	Migraine	4	1
	Bell’s palsy	3	0
	Fatigue	2	1
	Subdural hematoma	1	0
	Dementia	1	0
	Vertigo	1	5
	Syncope	0	3
	Infection	0	2
	Delirium	0	2
	Transitory Global Amnesia	0	1
	Opsoclonus Syndrome	0	1

**Table 3:** Agreement Between PreHAST Result and Consultant’s diagnosis

Consultant´s diagnosis		
Test result	Stroke or TIA	Non-stroke
PreHAST result		
Positive	26	26
Negative	0	17

Patients with deficit in any PreHAST item are defined as positive

The pilot study showed a sensitivity of 100% (95%CI; 87-100%) and a specificity of 40% (95%CI; 25-56%) for Stroke/TIA when a positive score in any PreHAST item (PreHAST score 1–19 points) was found after prehospital assessment in patients with suspected stroke. Furthermore, the positive - and negative predictive value was 50% and 100%, respectively, for a positive PreHAST score.

**Fig. 2:**
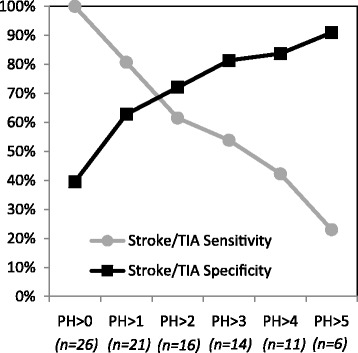
Sensitivity and Specificity for different PreHAST cut-off values Number of stroke/TIA patients with PreHAST scores (PH) higher than the given cut-off value are shown in parentheses

The discriminatory capacity for different cut off values for the PreHAST scores is illustrated in Fig. 2. The ROC analysis showed an area under the curve (AUC) of 0.77 (95%CI; 0.66-0.88).

**Fig. 3:**
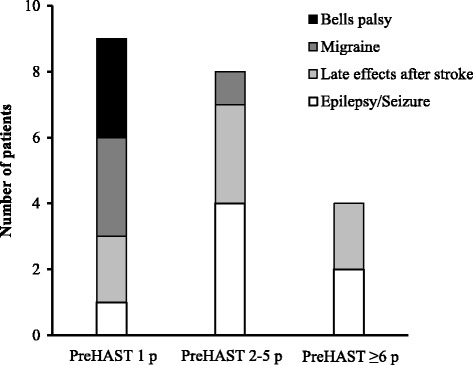
Stroke mimics with positive PreHAST scores The distribution of PreHAST scores in the subgroup of Non stroke patients (stroke mimics) who showed deficit at PreHAST evaluation (positive PreHAST scores). The four most frequent stroke mimic diagnosis with positive PreHAST scores is shown in different shadows of grey bars according to the PreHAST score category

Patients with Non-stroke diagnoses who showed deficit at PreHAST evaluation and thereby had positive PreHAST scores are shown in Table 2. The four most frequent Non-stroke diagnoses with PreHAST positive scores were epilepsy/seizure, late effect after stroke, migraine and Bells palsy. The distribution of the PreHAST scores among those most frequent Non-stroke diagnoses is shown in Fig. 3. Patients with Bells palsy or migraine showed low PreHAST scores, while if the final diagnosis was epilepsy/seizure and in patients with deficit after prior stroke (late effect after stroke) often had PreHAST scores between 2–5 points or even higher scores.

In the post-study survey, the ambulance staff reported PreHAST easy to execute and estimated the test time to be 2–3 min.

The correlation between the NIHSS and simulated PreHAST scores in the separate thrombolysis registry showed a correlation coefficient of 0.92 (95%CI 0.89-0.94)

## Discussion

PreHAST is a new screening test of typical stroke symptoms based on items from NIHSS, adapted for ambulance services, requiring 2–3 min to execute.

Los Angeles Prehospital Stroke Screen (LAPSS), Melbourne Ambulance Stroke Screen (MASS), Recognition of Stroke in the Emergency Room (ROSIER) and other prehospital stroke scales have balanced criteria for eligibility in favour of high specificity, i.e. excluding patients with suspected seizures (LAPSS, MASS, ROSIER) and age < 45 years (LAPSS, MASS) [3]. This may be unfortunate as seizure could be a sign of stroke and stroke may also well occur in the young. In FAST/CPSS, evaluation is limited to evaluation of facial palsy, arm paresis and speech disturbances. Patients with isolated hemianopia, neglect or leg paresis are therefore not captured, which could delay recognition and impede treatment.

PreHAST screens for a broad range of stroke symptoms including most key components of NIHSS. There is indirect evaluation of neglect and Wernicke’s aphasia built in the examination technique for the visual field, sensory and commands items. In our pilot, PreHAST identified all patients with stroke/TIA when stroke was suspected by ambulance personnel. However, patients with mild or unusual symptoms of stroke may be missed by the test. The calculated sensitivity between 87 and 100% is based on single centre observation in a small material and the diagnostic accuracy has to be confirmed in a larger study also including multiple centres. Furthermore, we did not include testing of interrater agreement in the design of our pilot study. Further studies will need to include this important element in the development of a new scale.

The high sensitivity for PreHAST could be at the expense of lower specificity. Stroke mimics are difficult to exclude from stroke triage and further investigation are sometimes necessary to exclude stroke. Patients without deficit according to PreHAST often had unspecific diagnosis such as vertigo or syncope, but were included by the ambulance staff, maybe to avoid missing vertebrobasilar stroke. A higher PreHAST score implied more specific pattern of symptoms for stroke, e.g. isolated dysarthria may be stroke but could have several other causes, whereas a pattern of dysarthria combined with hemianopia and left sided hemiparesis makes stroke more plausible. Accordingly, our study showed increasing specificity with higher PreHAST scores. Epilepsy/seizure, sequelae after prior stroke (late effect after stroke) and migraine regularly raises suspicion of stroke in the acute phase [14, 15] and were the most frequent stroke mimics, showing deficit after PreHAST evaluation in our study. To exclude patients with seizure at debut, prior stroke or known migraine from acute stroke triage could lead to decreased “over triage”, but with a risk of excluding true stroke patients from acute stroke treatment. Prior stroke is a risk factor of stroke, seizure could be the first sign of severe stroke and aura like symptoms could also be signs of a stroke [15]. A telephone or tele medicine support line to a stroke experienced physician for the ambulance staff [16] could in selected cases be of help but to a prize of time loss to transportation and a risk to overestimate the probability of stroke mimics. In patients with more severe deficit and a higher probability of large vessel occlusion it may be justified to weigh in the higher probability of stroke mimics in some patient groups before a decision of a change to another triage to minimize time to e.g. endovascular treatment is taken.

The Los Angeles Motor Scale (LAMS) [17], the Rapid Arterial oCclusion Evaluation (RACE) scale [18], the Cincinnati Prehospital Stroke Severity Scale (CPSSS) [19] and Prehospital Acute Stroke Severity Scale (PASS scale) [20] are other prehospital scores designed to grade stroke severity. Those are developed to identify patients with large vessel occlusion and are not intended for stroke screening in general. Furthermore, only the RACE scale has been evaluated in prehospital settings. The advantage of PreHAST is the dual purpose, both showing high sensitivity for stroke in general and grading stroke severity.

During 2017 we will start a multicenter triage study to evaluate the usefulness of PreHAST to identify thrombectomy candidates in the prehospital setting.

## Conclusions

PreHAST is a comprehensive test of typical stroke symptoms adapted for ambulance services with a high sensitivity for stroke. The grading system, similar to NIHSS, gives the test a quality with increasing specificity with higher scores. The properties of PreHAST, with the dual purpose of stroke screening in general and grading stroke severity, have the potential to be an important part in a prehospital triage system to identify patients for acute stroke treatments.

## Abbreviations

AUC: Area under the curve
CPSS: Cincinnati Prehospital Stroke Scale
CPSSS: Cincinnati Prehospital Stroke Severity Scale
FAST: Face Arm Speech Test
LAMS: The Los Angeles Motor Scale
LAPSS: Los Angeles Prehospital Stroke Screen
LOC: Level of consciousness
MASS: Melbourne Ambulance Stroke Screen
NIHSS: National Institute of Health Stroke Scale
PreHAST: PreHospital Ambulance Stroke Test
RACE: Rapid Arterial oCclusion Evaluation
RETTS: Rapid emergency triage and treatment system
ROC: Receiver Operating Characteristic
ROSIER: Recognition of Stroke in the Emergency Room
